# Primary Care Residents Lack Comfort and Experience with Alcohol Screening and Brief Intervention: A Multi-Site Survey

**DOI:** 10.1007/s11606-015-3184-y

**Published:** 2015-02-10

**Authors:** Kristy Barnes Le, J. Aaron Johnson, J. Paul Seale, Hunter Woodall, Denice C. Clark, David C. Parish, David P. Miller

**Affiliations:** 1 Department of Internal Medicine, Wake Forest School of Medicine, Medical Center Boulevard, Winston-Salem, NC USA; 2 Wake Forest School of Medicine, Winston-Salem, NC USA; 3 Georgia Regents University, Augusta, GA USA; 4 Mercer University School of Medicine & Medical Center of Central Georgia, Macon, GA USA; 5 AnMed Health, Anderson, SC USA

**Keywords:** alcohol screening, SBIRT, residency training, primary care screening, brief intervention, alcohol SBI

## Abstract

**BACKGROUND:**

Approximately one in six adults in the United States (U.S.) binge drinks. The U.S. Preventive Services Task Force recommends that primary care physicians screen patients for such hazardous alcohol use, and when warranted, deliver a brief intervention.

**OBJECTIVE:**

We aimed to determine primary care residents’ current practices, perceived barriers and confidence with conducting alcohol screening and brief interventions (SBI).

**DESIGN:**

This was a multi-site, cross-sectional survey conducted from March 2010 through December 2012.

**PARTICIPANTS:**

We invited all residents in six primary care residency programs (three internal medicine programs and three family medicine programs) to participate. Of 244 residents, 210 completed the survey (response rate 86 %).

**MAIN MEASURES:**

Our survey assessed residents’ alcohol screening practices (instruments used and frequency of screening), perceived barriers to discussing alcohol, brief intervention content, and self-rated ability to help hazardous drinkers. To determine the quality of brief interventions delivered, we examined how often residents reported including the three key recommended elements of feedback, advice, and goal-setting.

**KEY RESULTS:**

Most residents (60 %, 125/208) reported “usually” or “always” screening patients for alcohol misuse at the initial clinic visit, but few residents routinely screened patients at subsequent acute-care (17 %, 35/208) or chronic-care visits (33 %, 68/208). Only 19 % (39/210) of residents used screening instruments capable of detecting binge drinking. The most frequently reported barrier to SBI was lack of adequate training (54 %, 108/202), and only 21 % (43/208) of residents felt confident they could help at -risk drinkers. When residents did perform a brief intervention, only 24 % (49/208) “usually” or “always” included the three recommended elements.

**CONCLUSIONS:**

A minority of residents in this multi-site study appropriately screen or intervene with at-risk alcohol users. To equip residents to effectively address hazardous alcohol use, there is a critical need for educational and clinic interventions to support alcohol-related SBI.

## INTRODUCTION

Approximately one in six adult Americans binge drink and 4–5 % meet diagnostic criteria for alcohol dependence.^1^–^5^ For young adults aged 18–24 years, rates of binge drinking are even higher and exceed 25 %.^1^ Recently, binge drinking has been highlighted in the media after the Centers for Disease Control and Prevention (CDC) reported binge drinking rates in adult women and high school girls of 12.5 % and 19.8 %, respectively.^6^, ^7^ While medical school curricula have traditionally focused on alcohol dependence, the majority of alcohol-related death and morbidity is due to binge drinking (or acute alcohol misuse) and not chronic misuse.^8^ Unfortunately, physicians often fail to detect binge drinking in patients, despite its high prevalence.^9^–^15^

Alcohol screening and brief intervention (SBI) has been shown to be an effective tool for detecting and reducing unhealthy alcohol use, which includes both binge drinking and alcohol use disorders.^16^–^19^ In published randomized controlled trials, brief interventions decrease weekly alcohol consumption by 13–34 %.^20^ Since 2004, the U.S. Preventive Services Task Force (USPSTF) has recommended that all primary care practices incorporate SBI to detect and intervene with binge drinkers, defined as individuals who exceed the National Institute on Alcohol Abuse and Alcoholism (NIAAA) recommended daily alcohol limits.^3^, ^21^, ^22^ However, SBI is often not performed.^9^, ^13^, ^14^ Despite these recommendations, a recent report from the CDC found that only one in six adults reports discussing alcohol use with their health care provider.^23^

Clinicians’ lack of confidence in assessing alcohol use and providing brief advice is a significant barrier to SBI practice.^24^ Research indicates that low screening and intervention rates correlate with lack of training and low clinician self-efficacy.^25^–^27^ Although primary care residents commonly encounter patients with alcohol misuse, it is unknown how residents identify or intervene with these patients. Likewise, while residents lack confidence in addressing patients’ unhealthy alcohol use, the factors influencing confidence are unknown.^28^ To inform the development of a training program in Screening, Brief Intervention and Referral to Treatment (SBIRT) for primary care residencies, we conducted a multi-site survey to determine residents’ current practices, perceived barriers and confidence with conducting alcohol SBI.

## METHODS

### Study Setting and Participants

This cross-sectional survey study was conducted by the Southeastern Consortium for Substance Abuse Training (SECSAT), a consortium established to develop a comprehensive residency curriculum in SBIRT for alcohol and drug misuse (www.SBIRTonline.org). As of December 2012, the consortium included three internal medicine residency programs and three family medicine residency programs located in Georgia, South Carolina, and North Carolina. We invited all residents enrolled in these six training programs to participate. The Institutional Review Boards of all participating institutions approved the study, and all participants gave written informed consent.

### Survey Instrument

Between March 2010 and December 2012, before the SBIRT curriculum was introduced at each site, we asked all residents to complete a paper-based survey to assess their attitudes, beliefs, and current practice patterns pertaining to screening and intervening with hazardous drinkers. The survey was based on a previously validated instrument,^28^ pilot tested by faculty in the programs, and revised through consensus discussion. The survey required approximately 10 to 15 minutes to complete and contained 83 items, which were a mixture of multiple-choice questions, yes/no items, and Likert scale items. The survey assessed: 1) current practices and attitudes regarding screening and intervening with hazardous drinkers, 2) current practices and attitudes regarding screening and intervening with substance abusers, 3) prior training received, and 4) participant demographic information. We assigned all participants study numbers to protect confidentiality.

### Outcome Measurement

For this study, we focused on residents’ responses to the hazardous drinking items. Our main outcomes of interest were how often residents screened patients for alcohol misuse, their methods used for screening patients, their perceived barriers to discussing alcohol use, their perceived ability to help hazardous drinkers, the quality of brief interventions performed by residents, and characteristics that may make them more confident with conducting SBI. We assessed residents’ self-perceived ability to help hazardous drinkers by asking them how confident they are to “help [their] at-risk patients cut down or quit using alcohol.” Residents responded along a 5-point Likert scale ranging from 1 (Not at all confident) to 5 (Extremely confident). For analytical purposes, we transformed this measure into a binary variable, by considering any response of 4 or greater as “confident” and responses from 1 to 3 as “not confident.”

We defined a brief intervention as any discussion with a patient about their at-risk alcohol use. According to the USPSTF systematic review of the brief intervention evidence, effective interventions include at a minimum feedback, advice and goal-setting.^20^ Feedback refers to informing the patient of their level of risk based on their drinking patterns, while advice consists of giving medical recommendations to cut back or quit drinking. Along with the clinician, the patient then sets a goal for alcohol reduction to be reviewed at a follow-up visit. We asked residents how often their discussions about alcohol misuse included each of these three elements (feedback, advice, and goal-setting). Possible responses included “never”, “sometimes,” “about half the time,” “usually,” and “always.”. We assigned one point to each element the residents reported "usually" or "always" including in an intervention, yielding an ordinal intervention quality score ranging from 0 (no elements included) to 3 (all elements included).

### Data Analysis

Analyses were conducted using SPSS version 20.0 (IBM. Armonk, NY). In addition to frequency distributions, we used analysis of variance and chi-square tests, where applicable, to identify differences in means or proportions by resident demographics. To determine which factors were associated with residents’ confidence in their ability to help hazardous drinkers cut down on or quit using alcohol, we created a multivariate logistic regression model with confidence (as defined above) as the dependent variable and resident demographic factors as covariates: resident age (< 30 vs. 30 or older), race (white vs. nonwhite), country of birth (United States vs. other), religious affiliation (none, Christian, non-Christian), family history of substance abuse (yes vs. no), type of training program (Family Medicine vs. Internal Medicine), and year of training. All statistical tests were two-sided with an alpha of 0.05.

## RESULTS

### Participant Demographics

We invited all 244 residents in the six residency programs to participate, and 210 residents provided informed consent and completed the survey (response rate 86 %). Individual response rates from the six programs ranged from 78 % to 100 %. Table 1 summarizes the demographic characteristics of the respondents. Approximately 60 % of residents were from Internal Medicine training programs and 40 % from Family Medicine training programs. Half (48 %) were in their PGY1 year of training, 80 % were born in the United States, and 61 % were 30 years old or younger.

**Table 1.** Characteristics of Resident Study Sample

Resident Characteristics	n ( %)
Training Program, *n of 210 responders*	
Family Medicine	82 (39 %)
Internal Medicine	128 (61 %)
Residency Year, *n of 208 responders*	
PGY1	99 (48 %)
PGY2	71 (34 %)
PGY3	38 (18 %)
Age < 30, *n of 206 responders*	126 (61 %)
U.S. Born, *n of 206 responders*	164 (80 %)
Hispanic/Latino, *n of 206 responders*	10 (5 %)
Race,* n of 204 responders*	
African-American	17 (8 %)
Asian	34 (17 %)
White	126 (62 %)
Other	27 (13 %)

### Screening Instruments and BI Elements Used

**Figure 1.**
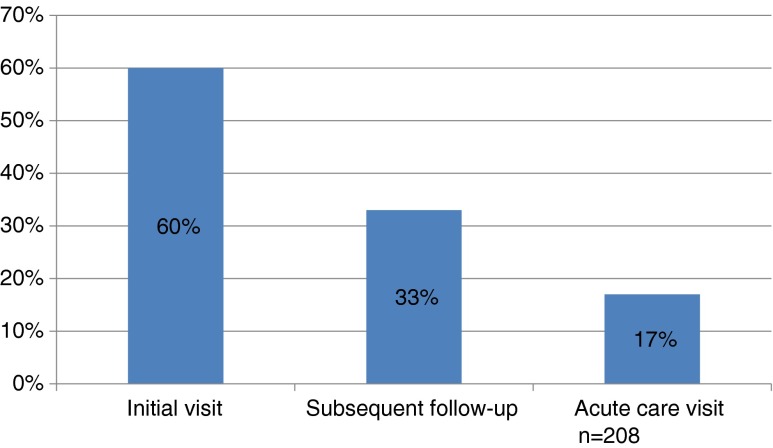
Percentage of residents who “usually” or “always” screen patients for at-risk drinking, by visit type.

**Figure 2.**
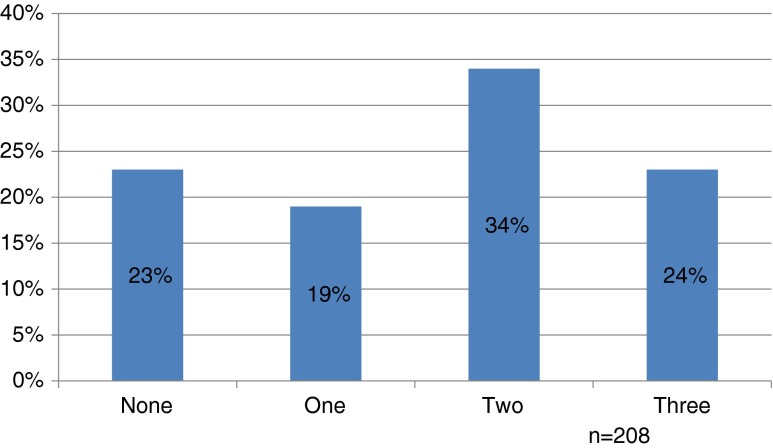
Number of key elements residents “usually” or “always” include in brief interventions. Key elements = feedback, advice, and goal-setting.

The majority of screening for hazardous alcohol use (60 %) occurred at the initial clinic visit (Fig. 1). Only one-third of residents (33 %) reported screening for alcohol misuse at subsequent follow-up visits, while fewer than one in five (17 %) reported regularly screening at acute-care visits where the consequences of hazardous drinking (such as injuries) are most likely to present. The most common screening instruments used were those designed to detect alcohol use disorders, and not binge drinking, namely the CAGE questionnaire (used by 63 % of residents) and Quantity/Frequency questions (used by 48 % of residents) (Table 2). In contrast, few residents (19 %, 39/210) used screening items that could detect binge drinking, such as the AUDIT, AUDIT-C, or the Single Alcohol Screening Questions (SASQ).^29^–^31^ When a brief intervention was performed, only 24 % of residents usually or always included the three recommended elements of feedback, advice, and goal-setting. In addition, 23 % of residents included none of these elements (Fig. 2).

**Table 2:** Proportion of Residents Who Report “Usually or Always” Using a Given Alcohol Screening Instrument (n=210)

Screening Instrument Used	n ( %)
CAGE	133 (63 %)
Quantity/Frequency Questions	100 (48 %)
Any instrument capable of detecting binge drinking	39 (19 %)
Single-item Alcohol Screening Question	34 (16 %)
AUDIT or AUDIT-C	7 (3 %)

*Some residents reported using more than one instrument

AUDIT = Alcohol Use Disorders Identification Test

AUDIT-C = Alcohol Use Disorders Identification Test—Consumption

### Barriers to SBI

Few residents felt confident or successful helping patients with hazardous drinking. Only 21 % (43/208) of respondents felt confident they could help their hazardous drinkers cut down or quit using alcohol, and only 17 % (35/208) thought they had been successful in doing so in the past. Residents indicated a number of key barriers to discussing alcohol use (Table 3). The most frequently reported barriers were lack of adequate training (53 %), the belief that talking with patients is unlikely to make a difference (44 %), and being too busy (39 %).

**Table 3.** Proportion of Residents Who Report a Barrier Moderately or Very Important

Barriers to Screening	n ( %)
Do not have adequate training *(n of 202)*	108 (53 %)
Talking with a patient about alcohol unlikely to make a difference *(n of 204)*	89 (44 %)
Too busy to have time to talk about alcohol with patients *(n of 204)*	79 (39 %)
Little financial reimbursement *(n of 204)*	64 (31 %)
Discussing alcohol is uncomfortable *(n of 204)*	58 (28 %)
Discussing alcohol threatens a good doctor–patient relationship *(n of 204)*	55 (27 %)

U.S. born residents were less likely to express confidence in their ability to help patients cut down or quit using alcohol than their non-U.S. born counterparts (17 % vs. 36 % respectively, p= 0.01). This association remained significant in multivariable analysis (OR 0.23; 95 % CI 0.08–0.70). Compared to residents who self-identified as Christian, residents reporting no religious affiliation expressed less confidence (OR 0.33; 95 % CI 0.11–0.99). No other demographic or personal factors were associated with residents’ confidence in the unadjusted or adjusted analyses. (Table 4)

**Table 4.** Association of Resident Characteristics With Confidence in Helping at-Risk Patients Cut Down or Quit Using Alcohol

Resident Characteristic	Confident helping at-risk drinkers*	
	n ( %)	Adjusted OR (95 % CI)^†^
Race		
Nonwhite, n=78 (ref)	19 (24 %)	1
White, n=124	21 (17 %)	0.99 (0.38–2.7)
Country of birth		
Outside United States, n=42 (ref)	15 (36 %)‡	1
United States, n=162	27 (17 %)	0.23 (0.08–0.70)
Age		
< 31, n=125 (ref)	21 (17 %)	1
31 or older, n=79	20 (25 %)	1.9 (0.89–4.2)
Religious affiliation		
Christian, n=122 (ref)	25 (20 %)	1
Nonchristian, n=31	8 (26 %)	0.85 (0.24–3.0)
None, n=46	8 (17 %)	0.33 (0.11–0.99)
Family history of drug/alcohol problem		
Yes, n=69 (ref)	17 (25 %)	1
No, n=137	25 (18 %)	0.47 (0.20–1.1)
Residency Program		
Family Medicine, n=82 (ref)	19 (23 %)	1
Internal Medicine, n=126	24 (19 %)	0.66 (0.30–1.4)
Residency Year		
PGY1, n=98 (ref)	21 (21 %)	1
PGY2, n=71	16 (23 %)	1.5 (0.65–3.4)
PGY3, n=37	5 (14 %)	0.50 (0.15–1.6)

* Residents reporting feeling “very” or “extremely” confident in their ability to help hazardous drinkers cut down or quit

† Adjusted OR from multivariable logistic regression model including all listed resident characteristics as covariates

‡ p < 0.01, Pearson Chi-Square test

Overall, residents reported receiving a mean of 9.8 hours of alcohol training over their careers (95 % CI 6.7–13.0 hours). Interestingly, the mean number of reported training hours received did not differ by year in residency (10.1 hours for PGY1, 10.9 hours for PGY2, 7.2 hours for PGY3; p = 0.716), suggesting that residents perceived most of the training to have occurred in medical school and not during residency. Additionally, the number of training hours reported did not correlate with their confidence in performing SBI.

## DISCUSSION

To our knowledge, this is the first study to examine in detail how residents are screening and intervening with patients with alcohol misuse. In this multi-site study, we found residents are using the wrong screening instruments at the wrong times and delivering suboptimal interventions. Over 80 % of residents are using screening instruments that will miss binge drinking and fail to screen patients at acute care visits where the consequences of binge drinking (i.e., falls, sprains, risky sexual encounters) are most likely to present. When residents do discuss hazardous drinking with patients, only 24 % of residents include the three key recommended elements of an effective brief intervention. Given these findings, it is not surprising that few residents are confident they can help patients reduce their hazardous drinking.

We found that only two demographic factors were associated with increased confidence in performing alcohol SBI: country of birth and religious identity. Foreign-born residents are significantly more confident in their ability to help hazardous drinkers than U.S. born residents. Although our survey did not ask participants to identify their medical school, slightly more than half of residents in our consortium who were born outside the U.S. also completed medical school training outside the U.S. It is unclear whether this difference stems from differences in cultural perceptions of alcohol use, differences in educational systems, or differences in prior alcohol training. Additionally, residents who identified themselves as Christian were also significantly more confident they could help their patients cut down or quit drinking than their counterparts without religious affiliation. This finding raises the question whether particular religious views about alcohol translate into confidence in discussing the topic.

Our study reveals a large need to improve alcohol-related training and screening processes in primary care residencies. Over half of residents reported that they lacked adequate training to intervene with hazardous drinkers. Although the average resident reported having received 9.8 hours of training on this topic, the amount of training received did not vary by residency year, suggesting that the majority of training was occurring during medical school and not residency. Furthermore, our finding that even third year residents lacked confidence in their ability to counsel patients about hazardous alcohol use indicates that clinical experience alone is insufficient for achieving this skill.

Previously published curriculum surveys have confirmed that residency teaching related to substance abuse is limited in family medicine and internal medicine residency programs.^32^, ^33^ In our study, the number of hours of substance abuse training received was unrelated to resident confidence, suggesting the quality of training is more important than the quantity. Indeed, prior research indicates educational sessions incorporating interactive components such as role plays are more effective than didactic lectures.^34^ Giving learners the opportunity to observe and practice encounters improves their communication skills and confidence.^35^–^37^ In recent years, educators have stressed the importance of simulation in medical training,^38^ and others have developed virtual environments where learners can practice counseling and alcohol SBI skills.^39^–^41^ In brief, increasing residents’ competence and confidence with brief interventions requires letting them practice these skills in an educational environment.

Effective screening systems are also needed to combat the belief that discussing alcohol use with patients will not make a difference, a belief endorsed by almost half of residents in our study. Brief interventions are most effective in people with non-dependent binge alcohol use, rather than those with alcohol dependence.^42^ Unfortunately, the screening instruments most residents report using (the CAGE and Quantity/Frequency questions) will detect the harder-to-treat alcohol use disorders, but not the much larger proportion of patients who are binge drinkers. Accordingly, implementing a screening system that efficiently identifies binge drinkers should improve the outcomes residents see, increasing their confidence in their ability to help patients change their drinking habits.

Well-designed screening systems can also address the third-most common barrier reported by residents, being “too busy” to talk about alcohol. Clinics can effectively screen patients for harmful drinking during the patient rooming process, offloading the screening task from busy physicians. The initial questioning by the staff naturally opens up the conversation about alcohol, and may shorten the face-to-face time required for discussion with the physician. Furthermore, nurse-based screening can save providers additional time by removing the need for physicians to discuss alcohol consumption with the 75 % or more of patients who screen negative.

Since collecting this data, our SECSAT consortium has worked to develop a broad-based SBIRT curriculum that incorporates both physician training and clinic-based screening protocols to identify patients with any hazardous alcohol use, not only those with abuse or dependence. The clinics in our consortium now screen all primary care outpatients for at-risk drinking every 6–12 months. During check-in, nursing staff screen patients with two questions: “Do you ever drink beer, wine or liquor,” and if answered affirmatively, “How many times in the past year have you had more than X drinks in one day (where x=3 for women and x=4 for men)?” Most of our sites have incorporated these screening questions into their electronic medical records, including automatic prompts for nursing to screen patients at the indicated times. Nursing staff give any patient who screens positive the AUDIT, which the physician then reviews during the office visit. In a prior study, this process of incorporating screening with the nursing intake increased subsequent interventions by providers.^43^

Our SECSAT alcohol curriculum consists of 9 hours of training for residents, and defines at-risk drinking, explores the epidemiology and consequences of at-risk drinking, outlines the steps for screening and assessing risk, and teaches a motivational interview-based approach to the brief intervention, as well as other alcohol-related topics. The curriculum includes an initial 3-hour workshop that employs didactic information, along with video examples of interventions and role-play–based practice sessions. We have made our curriculum freely available on-line (www.SBIRTonline.org). In addition to our consortium, the Substance Abuse and Mental Health Services Administration (SAMSHA) has funded other SBIRT programs as part of their medical residency cooperative to help promote SBI as part of the residency curriculum.^44^

Our study’s high response rate (86 %) and our sampling of residents from six programs in three different states increase our confidence in the results. However, our study does have important limitations. Our findings rely on resident self-report to estimate both the frequency of brief interventions performed and the elements contained in those interactions. Because social desirability bias encourages resident physicians to overestimate both the number and quality of their interventions with hazardous drinkers, the actual frequency and quality of interventions performed is likely even lower than what we report. The wide confidence intervals around the association of many demographic factors with resident confidence indicate a relative lack of power for this subanalysis. While we found that only country of origin and religious affiliation were significantly associated with confidence in intervening with hazardous drinkers, other demographic factors may also be related.

In conclusion, we found that resident physicians in internal medicine and family medicine programs rarely use screening instruments designed to detect hazardous drinking, rarely screen patients at acute care or follow-up visits where the consequences of hazardous drinking are likely to present, and rarely perform effective brief interventions. These outcomes are accompanied by low resident confidence levels and a perceived need for more training. Given that third-year residents were no more confident than first-year residents in helping patients cut down or stop drinking, it becomes clear that simply accruing patient care experience does not provide adequate SBI skill. Research demonstrates that when systematic screening for mental health disorders is performed in primary care settings, physicians often act on the results. However, patients with alcohol misuse are less likely to receive a referral to mental health,^45^ highlighting the need to equip primary care physicians to perform SBI. To effectively address the high prevalence of hazardous drinking among U.S. adults, residency programs should incorporate SBI curricula that focus on both resident training and the development of appropriate clinic systems to support screening and brief intervention.
